# Molecular characterization and zoonotic potential of *Enterocytozoon bieneusi*, *Giardia duodenalis* and *Cryptosporidium* sp. in farmed masked palm civets (*Paguma larvata*) in southern China

**DOI:** 10.1186/s13071-020-04274-0

**Published:** 2020-08-08

**Authors:** Zhengjie Yu, Xi Wen, Xitong Huang, Ruohong Yang, Yaqiong Guo, Yaoyu Feng, Lihua Xiao, Na Li

**Affiliations:** 1 Center for Emerging and Zoonotic Diseases, College of Veterinary Medicine, South China Agricultural University, Guangzhou, 510642 Guangdong China; 2 Guangdong Laboratory for Lingnan Modern Agriculture, Guangzhou, Guangdong 510642 China

**Keywords:** *Enterocytozoon bieneusi*, *Giardia duodenalis*, *Cryptosporidium*, Masked palm civet, Zoonotic potential

## Abstract

**Background:**

Masked palm civets are known to play an important role in the transmission of some zoonotic pathogens. However, the distribution and zoonotic potential of *Enterocytozoon bieneusi*, *Giardia duodenalis* and *Cryptosporidium* spp. in these animals remain unclear.

**Methods:**

A total of 889 fecal specimens were collected in this study from farmed masked palm civets in Hainan, Guangdong, Jiangxi and Chongqing, southern China, and analyzed for these pathogens by nested PCR and DNA sequencing.

**Results:**

Altogether, 474 (53.3%), 34 (3.8%) and 1 (0.1%) specimens were positive for *E. bieneusi*, *G. duodenalis* and *Cryptosporidium* sp., respectively. Sequence analysis revealed the presence of 11 novel *E. bieneusi* genotypes named as PL1–PL11 and two known genotypes Peru8 and J, with PL1 and PL2 accounting for 90% of *E. bieneusi* infections. Phylogenetically, PL4, PL5, PL9, PL10 and PL11 were clustered into Group 1, while PL1, PL2, PL3, PL6, PL7 and PL8 were clustered into Group 2. Assemblage B (*n* = 33) and concurrence of B and D (*n* = 1) were identified among *G. duodenalis*-positive animals. Further multilocus genotyping of assemblage B has revealed that all 13 multilocus genotypes in civets formed a cluster related to those from humans. The *Cryptosporidium* isolate from one civet was identified to be genetically related to the *Cryptosporidium* bamboo rat genotype II.

**Conclusions:**

To the best of our knowledge, this first report of enteric protists in farmed masked palm civets suggests that these animals might be potential reservoirs of zoonotic *E. bieneusi* and *G. duodenalis* genotypes.
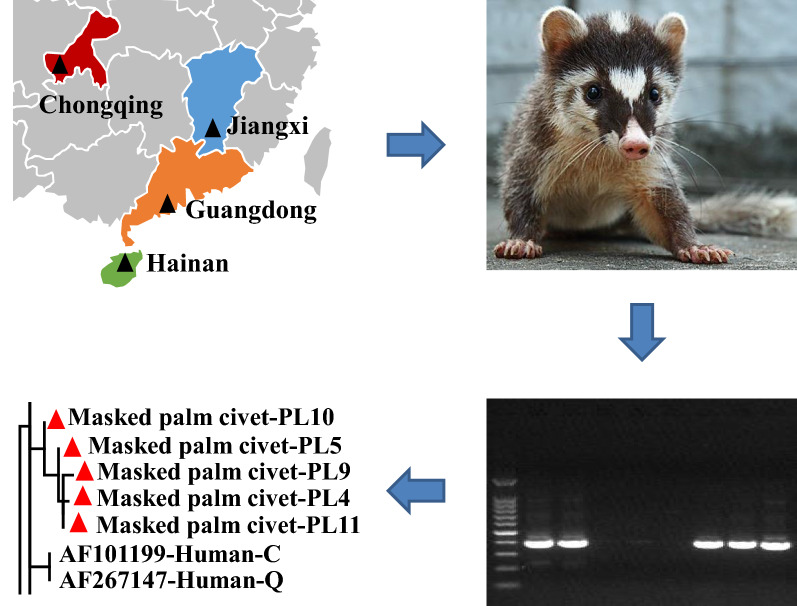

## Background

*Enterocytozoon bieneusi*, *Giardia duodenalis* and *Cryptosporidium* spp. are enteric pathogens in humans and various wild and domestic animals, causing diarrhea and other gastrointestinal symptoms. Humans can be infected by these pathogens through contact of contaminated fomites or ingestion of contaminated food or water (food-borne or water-borne transmission) [1–3].

The identification of genetic diversity in these pathogens has accelerated in recent years. Thus far, nearly 500 *E. bieneusi* genotypes have been identified, belonging to 11 phylogenetic groups with different host preferences, including the zoonotic Group 1 and host-adapted Groups 2–11 [2]. Similarly, eight distinct *G. duodenalis* assemblages of A–H with different host ranges have been identified by genetic characterization [4]. Among them, assemblages A and B are the most common causes of human giardiasis [5]. There are also nearly 40 recognized *Cryptosporidium* species and at least as many genotypes of unknown species status, most of which have host preference [3]. Recently, high-resolution multilocus genotyping (MLG) tools have been employed to elucidate the genetic heterogeneity of these pathogens in humans and animals [6–8].

Masked palm civets (*Paguma larvata*), belonging to the order Carnivora and family Viverridae, are wild mammals distributed widely in Asia [9]. In southern China, they are raised as new farm animals, as their meat is considered a culinary delicacy. In 2003, masked palm civets gained attention due to their potential involvement in the outbreak of severe acute respiratory syndrome (SARS), which originated from southern China and spread to over 30 countries [10]. In addition, results of other studies have suggested that they may play a role in the transmission of other zoonotic pathogens such as *Salmonella enterica*, *Campylobacter* spp., *Bartonella henselae* and *Toxoplasma gondii* [11–14]. Thus far, the occurrence and zoonotic potential of *E. bieneusi*, *G. duodenalis* and *Cryptosporidium* spp. in masked palm civets remain unclear.

This study was conducted to examine the prevalence, genetic identity and zoonotic potential of *E. bieneusi*, *G. duodenalis* and *Cryptosporidium* spp. in farmed masked palm civets in southern China.

## Methods

### Specimens

**Fig. 1:**
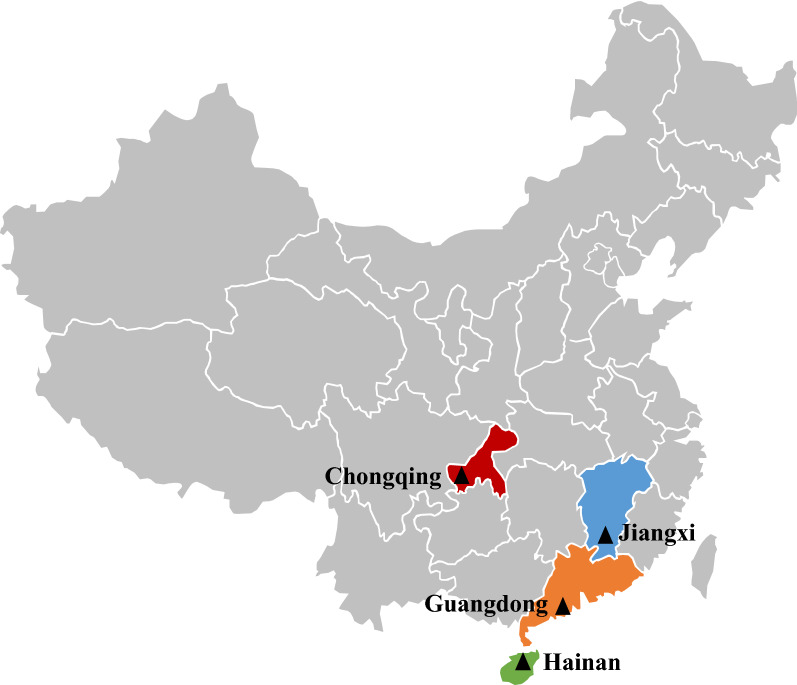
Locations (triangles) of four farms in southern China examined in the present study

From April 2018 to March 2019, a total of 889 fecal specimens were collected from masked palm civets on four commercial farms in Hainan, Guangdong, Jiangxi and Chongqing, southern China (Fig. 1). The management of animals on all four farms was similar, with the farm in Jiangxi being the largest in scale and having the longest history of over 20 years. The sanitary conditions of farms in Hainan and Chongqing were poor compared with the other two farms. Masked palm civets were kept in groups of 2–6 animals per cage, with some interactions with those in neighboring cages. Fresh fecal droppings from civets were collected on the ground under each cage, with one fecal specimen being collected from each cage for this study. The age of animals was divided into three groups: < 1 year (*n* = 469); 1–2 years (*n* = 129); and > 2 years (*n* = 291). All animals sampled in this study were clinically normal without obvious signs of diarrhea. Specimens were stored at 4 °C in 2.5% potassium dichromate before DNA extraction.

### DNA extraction

Prior to genomic DNA extraction, potassium dichromate was removed by washing 500 μl of fecal suspension three times with distilled water by centrifugation at 2000×*g* for 10 min. DNA was extracted from the sediment using the FastDNA Spin Kit for Soil (MP Biomedicals, Solon, OH, USA) as described [15]. The extracted DNA was stored at − 20 °C until being used in PCR analyses.

### PCR amplification

*Enterocytozoon bieneusi* was identified by nested PCR amplification of the internal transcribed spacer (ITS) of the rRNA gene [16]. *Giardia duodenalis* was detected by nested PCR targeting the triosephosphate isomerase (*tpi*), β-giardin (*bg*), and glutamate dehydrogenase (*gdh*) genes [17]. *Cryptosporidium* spp. were detected by PCR and sequence analyses of the small subunit (*SSU*) rRNA and further characterized by sequence analysis of the 60-kDa glycoprotein (*gp60*), 70-kDa heat-shock protein (*hsp70*) and actin genes [18–21]. Two replicates were used in PCR analysis of each target for each specimen. DNA of genotype PtEb IX from dogs, assemblage E from cattle, and *Cryptosporidium bovis* from cattle were used as positive controls in PCR analysis of *E. bieneusi*, *G. duodenalis* and *Cryptosporidium* spp., respectively, while reagent-grade water was used as the negative control.

### Sequence analysis

All positive secondary PCR products were sequenced bi-directionally on an ABI 3730 Genetic Analyzer (Applied Biosystems, Foster City, CA, USA). To determine the genetic identity of *E. bieneusi*, *G. duodenalis* and *Cryptosporidium* spp., sequences obtained were assembled using ChromasPro 2.1.6. (http://technelysium.com.au/ChromasPro.html), edited using BioEdit 7.1 (http://www.mbio.ncsu.edu/BioEdit/bioedit.html), and aligned with each other and reference sequences downloaded from GenBank using ClustalX 2.0.11 (http://clustal.org). Genotypes or subtypes of these pathogens were named according to the established nomenclature system [22, 23]. Maximum likelihood (ML) trees were constructed to infer the phylogenetic relationships among species or genotypes of these pathogens using MEGA 7.0.14 (http://www.megasoftware.net/). The general time-reversible model was used in substitution rate calculations and 1000 replicates were used in bootstrapping analysis.

### Statistical analysis

The Chi-square test was used to compare differences in the prevalence of pathogens among geographical locations or age groups. Differences were considered significant at *P* < 0.05.

### Nucleotide accession numbers

Representative nucleotide sequences generated in this study were deposited in the GenBank database under the accession numbers MT497888–MT497900 (*E. bieneusi*), MT487560–MT487588 (*G. duodenalis*), MT487589–MT487591 and MT779807 (*Cryptosporidium* sp.).

## Results

### Occurrence of *E. bieneusi*, *G. duodenalis* and *Cryptosporidium* sp

Occurrence of the three pathogens was determined based on the PCR positivity of at least one PCR replicate. Of the 889 fecal specimens collected from masked palm civets, 474 (53.3%) were positive for *E. bieneusi*, with infection rates ranging from 35.2% (172/489) to 86.1% (180/209) among the four farms sampled. Infection rates on the farms in Hainan (86.1%) and Chongqing (85.9%) were significantly higher than in Guangdong (46.2%; *χ*^2^ = 56.407 and 32.149, respectively; *P* < 0.0001) and Jiangxi (35.2%; *χ*^2^ = 152.05 and 76.11, respectively; *P* < 0.0001) (Table 1). By age, *E. bieneusi* infection rates in animals of < 1 year (61.0%; *χ*^2^ = 29.110, *P* < 0.0001) and 1–2 years (53.5%; *χ*^2^ = 5.734, *P* = 0.0166) were significantly higher than in animals of > 2 years (40.9%) (Table 1).

**Table 1:** Distribution of *Enterocytozoon bieneusi* and *Giardia duodenalis* genotypes in farmed masked palm civets in southern China by sampling location and age

Specimen	No. of specimens	*E. bieneusi*		*G. duodenalis*		Co-infection
		No. positive (%)	Genotype (*n*)	No. positive (%)	Genotype (*n*)	No. positive (%)
Hainan	209	180 (86.1)^a^	PL1 (95), PL2 (75)	8 (3.8)	B (8)	6 (2.9)
Chongqing	85	73 (85.9)^a^	PL1 (57), PL3 (11), PL2 (5)	7 (8.2)	B (6), B + D (1)	7 (8.2)
Guangdong	106	49 (46.2)	PL2 (30), PL1 (16), PL7 (1), PL8 (1)	14 (13.2)^c^	B (14)	13 (12.3)^e^
Jiangxi	489	172 (35.2)	PL1 (74), PL2 (59), PL4 (15), PL9 (5), PL6 (4), PL5 (3), PL10 (2), PL11 (2), J (1), Peru8 (1)	5 (1.0)	B (5)	5 (1.0)
< 1 year	469	286 (61.0)	PL1 (157), PL2 (87), PL3 (11), PL4 (10), PL9 (4), PL6 (4), PL5 (3), PL11 (2), PL10 (1), Peru8 (1)	13 (2.8)	B (12), B + D (1)	12 (2.6)
1–2 years	129	69 (53.5)	PL2 (35), PL1 (30), PL7 (1), PL8 (1)	14 (10.9)^d^	B (14)	13 (10.1)^f^
> 2 years	291	119 (40.9)^b^	PL1 (55), PL2 (47), PL4 (5), PL9 (1), PL10 (1), J (1)	7 (2.4)	B (7)	6 (2.1)
Total	889	474 (53.3)	PL1 (242), PL2 (169), PL4 (15), PL3 (11), PL9 (5), PL6 (4), PL5 (3), PL10 (2), PL11 (2), PL7 (1), PL8 (1), J (1), Peru8 (1)	34 (3.8)	B (33), B + D (1)	31 (3.5)

^a^*P* < 0.01, for Hainan and Chongqing in comparison with Guangdong and Jiangxi

^b^*P* < 0.05, for above 2 years-old in comparison with 1–2 years-old

^c^*P* < 0.01, for Guangdong in comparison with Hainan and Jiangxi

^d^*P* < 0.01, for 1–2 years old in comparison with under 1 year and above 2 years-old

^e^*P* < 0.01, for Guangdong in comparison with Hainan and Jiangxi

^f^*P* < 0.01, for 1–2 years-old in comparison with under 1 year and above 2 years-old

For *G. duodenalis*, 34 (3.8%) of the 889 specimens were positive based on the PCR positivity at any one of the three genetic loci. The highest infection rate was 13.2% (14/106) on the farm in Guangdong, followed by Chongqing (8.2%, 7/85), Hainan (3.8%, 8/209) and Jiangxi (1.0%, 5/489). The infection rate on the farm in Guangdong was significantly higher than in Hainan (*χ*^2^ = 9.525, *P* = 0.002) and Jiangxi (*χ*^2^ = 37.993, *P* < 0.0001) (Table 1). Additionally, animals of 1–2 years (10.9%) had significantly higher prevalence of *G. duodenalis* than those of < 1 year (2.8%; *χ*^2^ = 15.324, *P* < 0.0001) and > 2 years (2.4%; *χ*^2^ = 13.427, *P* = 0.0002) (Table 1).

Among the 889 specimens analyzed, only one (0.1%) from the farm in Hainan was positive for *Cryptosporidium* sp. This civet was co-infected with *E. bieneusi*. In addition, co-infection of *E. bieneusi* and *G. duodenalis* was detected in 31 other animals, with a significantly higher occurrence of co-infection of the two pathogens on the farm in Guangdong (12.3%) than in Hainan (2.9%; *χ*^2^ = 10.949, *P* = 0.0009) and Jiangxi (1.0%; *χ*^2^ = 33.793, *P* < 0.0001). By age, a significantly higher co-infection rate was detected in civets aged 1–2 years (10.1%) than < 1 year (2.6%; *χ*^2^ = 14.278, *P* = 0.0002) and > 2 years (2.1%; *χ*^2^ = 13.296, *P* = 0.0003) (Table 1).

### *Enterocytozoon bieneusi* genotypes

**Fig. 2:**
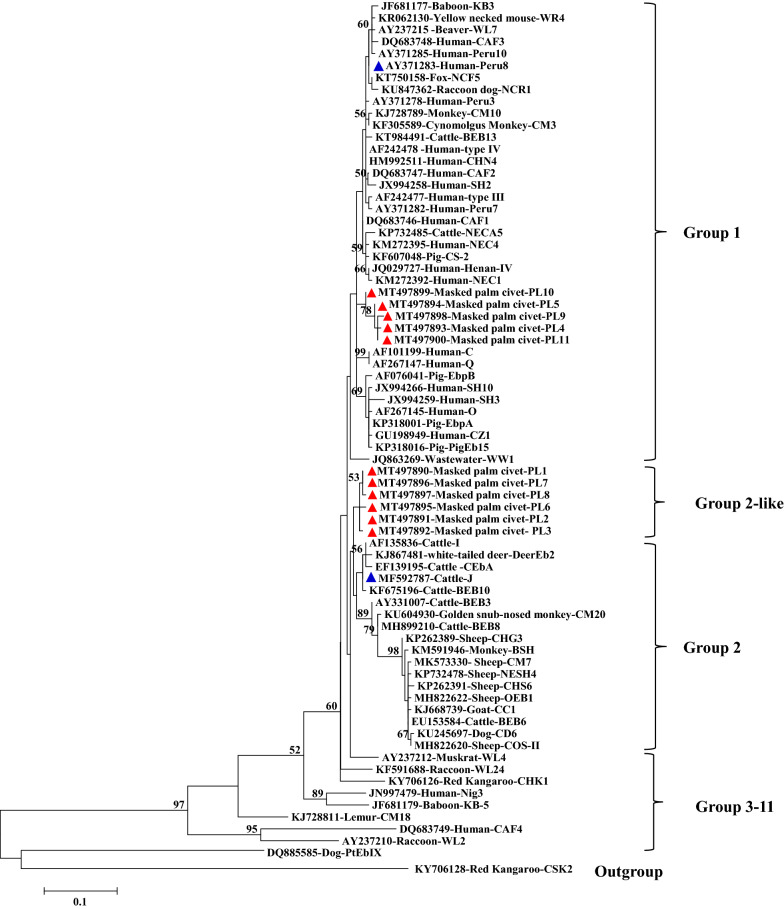
Phylogenetic relationship among *Enterocytozoon bieneusi* genotypes based on the maximum-likelihood analysis of the internal transcribed spacer of the rRNA gene. Bootstrap values greater than 50% from 1000 replicates are shown on the branches. Known and novel genotypes identified in this study are indicated by blue and red triangles, respectively

Sequence analysis of the ITS PCR products was successful for 457 of 474 *E. bieneusi*-positive specimens. The remaining 17 specimens generated sequences with underlying signals of mixed nucleotides, probably due to concurrence of more than one *E. bieneusi* genotype in each specimen. Altogether, 13 *E. bieneusi* genotypes were detected, including two known ones (Peru8 and J) and 11 novel genotypes (named as PL1–PL11) (Table 1). The latter were identified based on nucleotide sequence differences from known *E. bieneusi* genotypes. The ITS sequences of Peru8 in Group 1 and genotype J in Group 2 were identical to the GenBank reference sequences AY371283 and MF592787, respectively. Among the 11 novel *E. bieneusi* genotypes, PL4, PL5, PL9, PL10 and PL11 were phylogenetically placed in Group 1, while PL1, PL2, PL3, PL6, PL7 and PL8 formed a clade named as Group 2-like, which was mostly related to Group 2 (Fig. 2).

PL1 (53.0% or 242/457) and PL2 (37.0% or 169/457) were the two most prevalent genotypes (Table 1). PL1 was the dominant genotype on the three farms in Hainan, Chongqing and Jiangxi, while PL2 was the most common one on the farm in Guangdong. The highest genetic diversity was observed on the farm in Jiangxi, with 2 known and 8 novel genotypes. Several unique genotypes were detected on some of the farms, including PL4, PL5, PL6, PL9, PL10, PL11 on the farm in Jiangxi, PL7 and PL8 on the farm in Guangdong, and PL3 on the farm in Chongqing.

### *Giardia duodenalis* assemblages

Among the 34 *G. duodenalis*-positive specimens, assemblage B was the dominant genotype, being detected in 33 specimens. One specimen, however, had the concurrence of assemblages B and D. By genetic locus, 13 sequence types were present among the 28 assemblage B-positive specimens at the *tpi* locus, including 3 known ones and 10 new ones. The three known sequence types, i.e. MB8 (*n* = 8), MB7 (*n* = 3) and B14 (*n* = 1), were identical to GenBank reference sequences KF679746, KF679745 and KF679737, respectively. In contrast, the new sequence types B-PL01 to B-PL10 had 1 to 6 single nucleotide polymorphisms (SNPs) compared with the GenBank reference sequence KF679746.

At the *bg* locus, one known and two new sequence types were obtained among the 16 assemblage B-positive specimens. Bb-3 (*n* = 5) was identical to the GenBank reference sequence KJ888976, while the new sequence types of B-PL11 and B-PL12 had one SNP compared with the GenBank reference sequence KJ888976.

At the *gdh* locus, 10 new sequence types (B-PL13 ~ B-PL22) were detected among the 22 assemblage B-positive specimens, with 1–4 SNPs compared with the GenBank reference sequence LC430575.

### Multilocus genotypes of *G. duodenalis* assemblage B

**Fig. 3:**
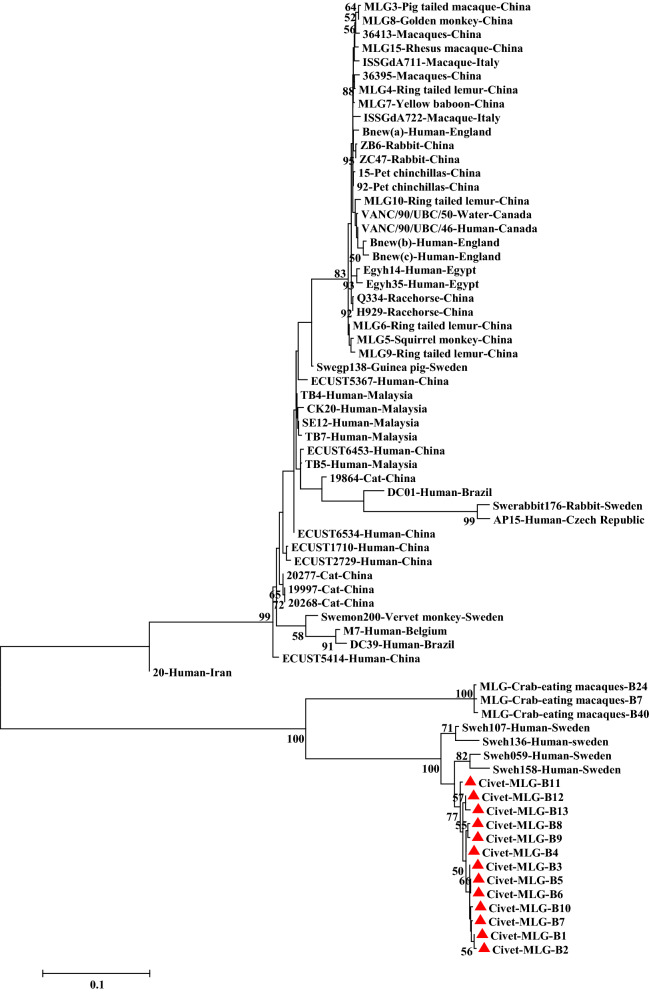
Phylogeny of multilocus genotypes (MLGs) of *Giardia duodenalis* assemblage B from this and previous reports based on the maximum-likelihood analysis of concatenated sequences of the *gdh*, *bg* and *tpi* loci. Bootstrap values greater than 50% from 1000 replicates are shown on the branches. MLGs identified in this study are indicated by red triangles

Of the 33 assemblage B-positive specimens, 13 were positive by PCR at all three loci, forming 13 MLGs (Civet-MLG-B1 to Civet-MLG-B13) based on the concatenated sequences of the *gdh*, *bg* and *tpi* loci. Phylogenetic analysis of these MLGs was conducted together with those from previous studies of assemblage B in humans and various animals [24–28]. It showed that all 13 MLGs from civets in this study formed a cluster, which was a sister group with MLGs from humans in Sweden (Fig. 3).

### *Cryptosporidium* genotype

**Fig. 4:**
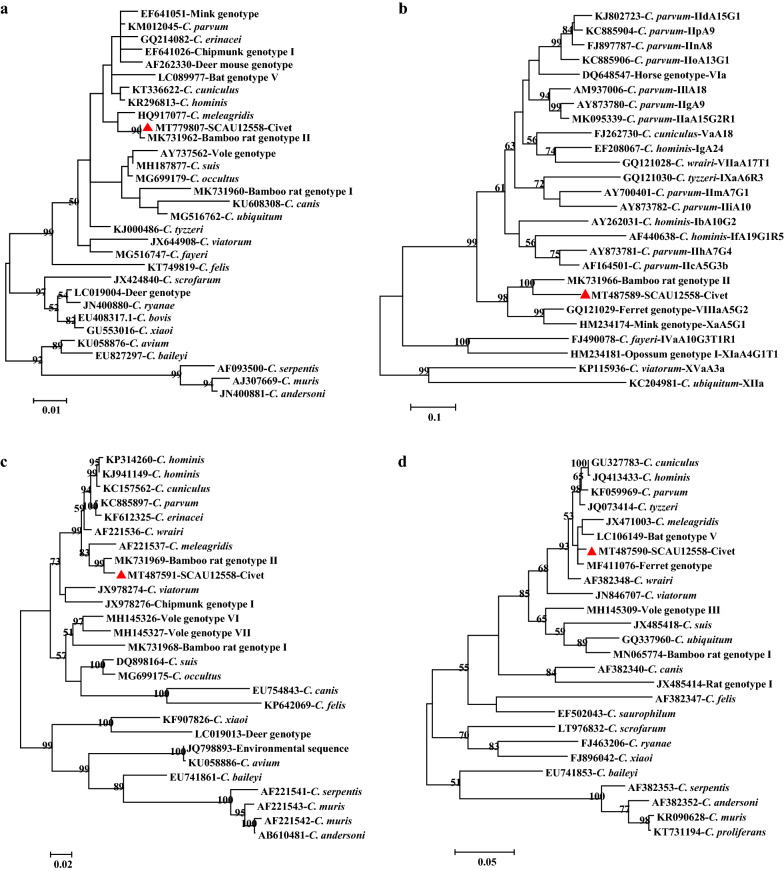
Phylogeny of *Cryptosporidium* spp. based on the maximum-likelihood analyses of the small subunit rRNA (**a**), 60-kDa glycoprotein (**b**), 70-kDa heat-shock protein (**c**) and actin (**d**) genes. Bootstrap values greater than 50% from 1000 replicates are shown on the branches. The *Cryptosporidium*-positive specimen detected in this study is indicated by red triangle

For the *Cryptosporidium*-positive specimen SCAU12558, DNA sequencing of PCR products of the *SSU* rRNA, *gp60* and *hsp70* genes revealed that it was phylogenetically related to the *Cryptosporidium* bamboo rat genotype II (Fig. 4). The *SSU* rRNA sequence generated had one SNP and three nucleotide insertions compared with the sequence from the bamboo rat genotype II (GenBank: MK731962). The *gp60* sequence generated had a maximum nucleotide identity of 83.0% to the bamboo rat genotype II (GenBank: MK731966), with extensive sequence differences in the non-repeat regions. The *hsp70* sequence generated had 17 SNPs and 1 nucleotide deletion with the bamboo rat genotype II (GenBank: MK731969), mostly over the repeat region at the 3′-end of the gene. Due to the absence of the actin gene sequence from the bamboo rat genotype II in the GenBank database, the actin sequence of SCAU12558 was found to be most similar to the *Cryptosporidium* ferret genotype (GenBank: MF411076) with 12 SNPs.

## Discussion

In this study, to the best of our knowledge, we report for the first time the occurrence and genetic identity of *E. bieneusi*, *G*. *duodenalis* and *Cryptosporidium* sp. in masked palm civets, with infection rates of 53.3%, 3.8% and 0.1%, respectively. Within the family Viverridae, several small carnivores genetically related to masked palm civets have been examined for these pathogens without much success. For example, a recent study in Spain reported the occurrence of an unknown *Cryptosporidium* genotype in one of six genets (*Genetta genetta*) examined for *Cryptosporidium* spp. and *G*. *duodenalis* [29]. Another study in the Philippines examined an Asian palm civet (*Paradoxurus hermaphroditus*) for *G*. *duodenalis* but did not detect this pathogen [30].

Results of the present study have revealed some differences in infection rates of *E. bieneusi* and *G. duodenalis* among farms and age groups. Significantly higher infection rates of *E. bieneusi* were observed on farms in Hainan (86.1%) and Chongqing (85.9%) than in Guangdong (46.2%) and Jiangxi (35.2%). This was likely due to the poor sanitary conditions of the two farms in Hainan and Chongqing, as the housing and management of animals were similar among the four farms. By age, a higher prevalence of *E. bieneusi* was obtained from young civets of < 1 year. This is in agreement with previous findings of age-associated occurrence of *E. bieneusi* infections in other farmed wild animals such as macaques, boars and squirrels [31–33]. In contrast, a significantly higher infection rate of *G*. *duodenalis* was observed on the farm in Guangdong, and masked palm civets of 1–2 years had the highest infection rate of *G*. *duodenalis*, suggesting that *E. bieneusi* and *G. duodenalis* are transmitted differently in these animals.

Results of the sequence analysis have demonstrated a high genetic diversity of *E. bieneusi* in masked palm civets. Altogether, 13 *E. bieneusi* ITS genotypes were identified in this study, with two novel genotypes PL1 and PL2 accounting for about 90% *E. bieneusi* infections in civets. These two ITS genotypes together with several other novel ones formed a clade related to Group 2, thus might be civet-adapted *E. bieneusi* genotypes. In comparison, the remaining novel genotypes PL4, PL5, PL9, PL10 and PL11 were phylogenetically clustered into the zoonotic Group 1. In addition, one civet was found positive for Peru8, which is a common zoonotic genotype in humans [2]. Thus, masked palm civets could carry human-pathogenic *E. bieneusi*.

High genetic heterogeneity of *E. bieneusi* was observed in masked palm civets on a large farm in Jiangxi. This farm is the largest among the four study farms and has been established for over 20 years. It has 10 of the 13 *E. bieneusi* genotypes identified in the study, including all six ITS genotypes (Peru8, PL4, 5, 9, 10 and 11) of the zoonotic Group 1. One explanation for the high heterogeneity is that genetic exchange might have occurred among ancestral types on this farm during the past 20 years [34]. The long history of the farm and high genotype diversity have probably facilitated the genetic exchange among *E. bieneusi* isolates and emergence of novel genotypes in farmed masked palm civets.

The zoonotic assemblage B appears to be the dominant *G*. *duodenalis* genotype in masked palm civets. In this study, all *G*. *duodenalis*-positive civets harbored assemblage B, with only one having concurrent infection of assemblages B and D. Assemblage B is the most common human-pathogenic genotype in both industrialized and developing countries [4, 35]. In animals, assemblage B has been recently reported as the dominant *G*. *duodenalis* genotype in farmed macaques, horses, donkeys, rabbits, chinchillas and bamboo rats, mostly in China [24, 36–43]. Within assemblage B found in this study, a high genetic heterogeneity was observed at the three genetic loci examined. Of the known subtypes, MB7 and MB8 at the *tpi* locus and Bb-3 at the *bg* locus were reported in non-human primates [27, 44], while B14 at the *tpi* locus was detected in urban wastewater previously [45]. In addition, phylogenetic analysis of the concatenated sequences from the three loci showed that the MLGs of assemblage B in civets formed a separate cluster, which confirms the previous suggestion on the likely occurrence of host adaptation within assemblage B [24, 27]. Nevertheless, the assemblage B MLGs from civets were genetically related to several MLGs from humans, indicating that they could have zoonotic potential.

The *Cryptosporidium* isolate from masked palm civets was found to be genetically related to the *Cryptosporidium* bamboo rat genotype II from bamboo rats in south-central China based on sequence analysis of the *SSU* rRNA, *gp60* and *hsp70* genes [46]. As only one fecal specimen was positive for *Cryptosporidium*, whether it is a native parasite of masked palm civets remains unclear. Further studies are needed to understand its host range.

## Conclusions

This report of the occurrence and identity of *E. bieneusi*, *G*. *duodenalis* and *Cryptosporidium* bamboo rat genotype II in farmed masked palm civets in southern China extends our knowledge on the genetic diversity and transmission of these pathogens in farmed wild animals. The presence of zoonotic *E. bieneusi* genotypes and *G. duodenalis* assemblage B in masked palm civets suggests that these animals might be potential reservoirs for human infections with these pathogens. Further studies involving extensive sampling of animals as well as farmworkers are needed to elucidate the role of newly domesticated wild animals in the epidemiology of human microsporidiosis, giardiasis and cryptosporidiosis.

## Data Availability

Data supporting the conclusions of this article are included within the article. Representative nucleotide sequences generated in this study were deposited in the GenBank database under the accession numbers MT487560-MT487591, MT497888-MT497900 and MT779807.

## Abbreviations

PCR: polymerase chain reaction
ITS: internal transcribed spacer
*tpi*: triosephosphate isomerase
*bg*: β-giardin
*gdh*: glutamate dehydrogenase
*SSU* rRNA: the small subunit rRNA
*gp60*: 60-kDa glycoprotein gene
*hsp70*: 70-kDa heat shock protein gene
ML: maximum likelihood
SNPs: single nucleotide polymorphisms
MLGs: multilocus genotypes
